# Interventions for cancer screening among Chinese Americans: A systematic review and meta-analysis

**DOI:** 10.1371/journal.pone.0265201

**Published:** 2022-03-16

**Authors:** Fang Lei, Ying Zheng, Eunice Lee

**Affiliations:** 1 School of Nursing, University of California at Los Angeles, Los Angeles, California, United States of America; 2 Shenzhen Nanshan Medical Group Headquarter, Shenzhen, China; University of California, San Francisco, UNITED STATES

## Abstract

**Background:**

Cancer is the leading cause of death among Chinese Americans (CAs). Although death rates of cancers can be significantly reduced by screening cancers at an early stage, cancer screening (CS) rates are low among CAs. Interventions on CS may increase the uptake rates of CS and help to decrease the death rates of cancers in CAs.

**Objectives:**

This study aims to summarize the intervention methods on CS among CAs and compare effects of various intervention methods on the outcomes of CS, including knowledge levels of CS, intentions to complete CS, and actual completions of CS.

**Methods:**

A systematic review and meta-analysis design was used. Keyword searching was conducted on PubMed, Google Scholar, PsycINFO, and CINAHL. Inclusion and exclusion criteria were applied. The PEDro scale was used to evaluate the quality of the studies. Data was analyzed using Review Manager Version 5.4 software. Random effect model and subgroup analyses were conducted.

**Results:**

The search yielded 13 eligible studies. All of the reviewed interventions were culturally tailored. Systematic review results were categorized by intervention delivery objects, intervention led, intervention contact, intervention types, and intervention focus according to group consensus. Meta-analysis results showed that the interventions on CS had a positive effect on all outcomes, including a 1.58 (95% CI, 1.17–2.14; P = 0.003), 1.78 (95% CI, 1.27–2.48; P = 0.0007), and 1.72 (95% CI, 1.22–2.42; P = 0.002) effect on knowledge of CS, intentions to complete CS, and completions of CS, respectively, compared to the control group. The subgroup analysis suggested that physician-led, individual-based, face-to-face client-focused interventions with multiple components increased CS among CAs, with the OR ranging from 1.60 (95% CI, 1.08–2.39; P = 0.02) to 3.11 (95%CI, 1.02–9.49; P = 0.05).

**Discussion:**

Interventions on CS significantly increased CAs’ knowledge of CS, intentions to complete CS, and completions of CS. Physician-led, individual-based, face-to-face client-focused interventions with multiple components should be utilized for CAs.

## Introduction

### Cancer mortality rates among Chinese Americans

In the United States, Chinese Americans are the largest Asian ethnic group, contributing to over one-fifth of the total Asian American population [1]. Cancer is the leading cause of death among Asian Americans, including Chinese Americans [2], with prostate cancer (8% for males), breast cancer (14% for females), colorectal cancer and lung cancer (about 8% and 27% for both genders) as the most common causes of cancer death in 2016 [3].

### Screening strategies for cancers

Cancer screening has been proven to be an effective way to detect cancers at an early stage and to reduce mortality rates [4]. For the most-commonly occurring five types of cancers, which include prostate cancer, cervical cancer, breast cancer, colorectal cancer, and lung cancer, early-detection methods can be utilized. In the United States, for men aged 55 to 69 years old, a prostate-specific-antigen (PSA) test every two years with physician’s recommendation is recommended to screen for prostate cancer, per U.S. Preventive Services Task Force (USPSTF)’s recommendation and the Cluster Randomized Trial of PSA Testing for Prostate Cancer Group’s report [5, 6]. In addition, according to the recommendation from USPSTF [7], women aged 21 to 65 years old should screen for cervical cancer regularly. With Papanicolaou (Pap) testing, eligible women should screen for cervical cancer every 3 years [8]. Also, women aged between 50 to 74 years old should get mammograms every two years [9]. Furthermore, the USPSTF recommends screening with a fecal occult blood test (FOBT) annually, sigmoidoscopy every 5 years, or colonoscopy every 10 years for average-risk individuals aged 50–75 years for colorectal cancer [10]; and annual screenings for lung cancer with low-dose computed tomography (LDCT) in adults aged 50 to 80 years who have a 20 pack-year smoking history (smoke 1 package of cigarettes per day for 20 years) and currently smoke or have quit within the past 15 years [11].

### Uptake rates of cancer screening among Chinese Americans

Although several health organizations have recommended high-risk populations (people who meet the criteria of the USPSTF recommendation of screening cancers) to screen for cancers regularly, compared to non-Hispanic whites, Chinese Americans were less likely to have ever been screened [12] or been up to date [13]. From 2000 to 2015, the colorectal cancer screening rate was the only one that increased among the uptake rates of breast, cervical, colorectal, and prostate cancers among US adults [14]. Among all ethnicities in the US, non-Hispanic Asian Americans generally reported the lowest cancer screening rate for all kinds of cancers [14]. Although cancer screening trends among Asian Americans lack report, cancer screening rates for Chinese Americans are generally lower than those for non-Hispanic whites and are even lower among those with limited English proficiency. During 2013 and 2014, rates for cervical cancer screening with the pap test among Chinese Americans and non-Hispanic whites in the United States were 65.8% vs. 82.8%, for breast cancer screening with mammograms were 65.6% vs. 68.9%, and for colorectal cancer screening with endoscopy/FOBT were 53.6% vs. 60.5% [15], respectively. Among older Chinese Americans, prior research also found that participation in early detection cancer screening was less likely, compared to other Americans [16, 17].

### Cancer screening interventions

To increase the uptake rate of cancer screening, interventions which aimed to increase community demand, community access, and provider delivery have been conducted. Several studies have been conducted to evaluate the effects of these interventions on the uptake rates of breast, cervical, and colorectal cancer screening [18, 19]. Researchers found that both client-focused interventions (e.g., client reminders [18, 19], outreach, education, navigation, and small media including videos or tailored or untailored printed materials, such as letters, brochures, pamphlets, flyers, or newsletters distributed by healthcare systems or community groups [18]) and provider-focused interventions (e.g., clinician reminders [19], face-to-face education of clinicians [19], and provider assessment and feedback Involving evaluation of provider performance in delivering or offering screening to clients and presenting providers with information about their performance in providing screening services [18]) seem to be effective in increasing the uptake rates of screening for cancers [18]. Also, researchers found that combinations of interventions were associated with greater increases compared to single components; and repeated interventions were associated with increased annual FBT completion [18].

### Outcomes of Knowledge of cancer screening, intention to screening cancers and completion of cancer screening

Participants’ uptake rates of cancer screenings were significantly related to their knowledge about screenings. Previous studies have revealed that knowledge promotes women’s participation in different kinds of cancer screenings [20–23]. A study conducted with participants aged 50–75 years old in South Carolina showed that higher level of knowledge was associated with a greater likelihood of having ever been screened for colorectal cancer (odds ratio [OR]: 1.05; 95% CI: 1.02–1.41; p < 0.001) [24]. Similarly, in the study conducted by Chen et al. [25] and the study conducted by Guo, Zhang, and Wu [26], results revealed that knowledge level influenced willingness towards and behaviors related to cervical cancer screening and breast cancer prevention intentions in Chinese women, respectively.

In addition, participants’ intentions to screening cancers were essential for them to complete cancer screening. Researchers found that participants who formed implementation intentions (e.g., the intentions motivate the individual to act, also the individual has developed strategies and plans that promote behavioral enactment [27]) were much more likely to complete screening, compared to the participants who didn’t form implementation intentions (92% vs. 69%) [28]. Evidence also suggests that implementation intentions attenuated the relationship between previous delay behavior and subsequent attendance for cervical cancer screening [28].

### Research question, purpose, and significance of the study

Intervention projects on cancer screening can increase the uptake rate of cancer screening among high-risk populations. Despite findings from previous intervention studies which provided information to increase cancer screening rates among the US population, these studies suggested a need for more studies to assess one-on-one education, group education interventions, etc. [18]. Furthermore, several systematic reviews and meta-analyses have been done to examine the effects of cancer screening interventions on the uptake rates of screening among the US population [18, 19]. However, to our best knowledge, no systematic review and meta-analyses have been done to examine the effects of cancer screening interventions on the uptake rates of cancer screening in Chinese Americans to date. With the supposition that the uptake of cancer screening could be impacted by culture, researchers have highlighted the importance of culture on behavior and indicated a need to assess culturally sensitive, theory-based interventions to encourage screening and reduce cancer-related health disparities [29]. From this point, a systematic review and meta-analysis to examine the effects of cancer screening interventions (e.g., culturally fitted interventions) on the uptake rate of cancer screenings among Chinese Americans is necessary.

The research questions aimed to be answered in this study were two-fold: (1) What intervention methods have been used for increasing cancer screening rates among Chinese Americans in the past ten years? and (2) Which intervention methods are effective and how effective are they? The purpose of this systematic review and meta-analysis is to investigate and summarize the intervention methods focusing on cancer screening among Chinese Americans and compare the effects of intervention methods on the outcomes of cancer screening, including the knowledge levels of cancer screening, intentions to complete cancer screening, and completions of cancer screening. This study will provide a comprehensive picture of the intervention programs which have been done on cancer screenings for Chinese Americans over the past ten years. It will also suggest an optimal way to increase cancer screening rates among Chinese Americans.

## Materials & methods

We conducted a systematic review and meta-analysis to explore the study aim, according to the Preferred Reporting Items for Systematic Reviews and Meta-analyses (PRISMA) guidelines.

### Data sources and searches

In this study, databases including PubMed, Google Scholar, PsycINFO, and CINAHL were searched. Keywords for searching were 1) Chinese Americans and 2) cancer screening related keywords, including cancer screening, mammogram, colonoscopy, FOBT, sigmoidoscopy, prostate-specific antigen, PSA, Pap, HPV, Cancer prevent*, lung cancer screening, low dose CT, and low dose computed tomography. For example, we used the Boolean search strategy: Chinese American* AND (cancer screening OR mammogram OR colonoscopy OR FOBT OR sigmoidoscopy OR prostate-specific antigen OR PSA OR Pap OR HPV OR Cancer prevent* OR lung cancer screening OR low dose CT OR low dose computed tomography) in the PubMed database to search the eligible literature. Equivalent words with similar meanings were also searched.

### Study selection and eligibility criteria

To exhaust the research articles that addressed interventions on cancer screening among Chinese Americans in the past ten years, we checked the titles of the articles first, then screened the abstract and text of the articles, and inspected references from the eligible articles for further inclusion. The inclusion criteria for selecting eligible articles were: 1) peer-reviewed articles, 2) intervention studies focusing on cancer screening among Chinese Americans, or minority populations including Chinese Americans, 3) published in the English language in the last ten years through June 20, 2021, and 4) with full text available. Studies were excluded if they were 1) study protocols or other informal articles (e.g., letter to editors, commentaries, etc.) without data supported; or 2) not meeting inclusion criteria.

### Data synthesis and study quality

The first and second author of this study did the initial searching in the databases separately. After identifying the eligible articles respectively, the authors had an in-depth discussion about which articles should be included and excluded. Disagreements were solved by consulting another researcher in the field. Information on the purposes, samples, study designs, methods, and results of the studies were exacted to a table of evidence to facilitate data analysis.

The methodological quality of each study was assessed using the PEDro scale [30]. The scale comprises a list of 11 criteria. Each criterion is valued by either a 0 (“No) or 1 (“Yes”), with only 10 of them used (item 2 to 11) to calculate the total score, yielding a maximum score of 10 points for each assessed study. The item 1 is used to evaluate studies’ external validity, which is not included when accessing studies’ PEDro score. Higher scores indicated superior methodological quality. Studies with a score lower than 4 are considered ‘poor’ quality, 4 to 5 are considered ‘fair’, 6 to 8 are considered ‘good’ and 9 to 10 are considered ‘excellent’ [30]. The agreement between the two reviewers was evaluated with the intraclass correlation coefficient (ICC).

### Data analysis

We used the Review Manager Version 5.4 software to conduct the meta-analysis. Random effect model was applied in the analysis. A range of exploratory post-hoc subgroup analyses were conducted to examine the effects of the intervention delivery objects, intervention led, intervention contact, intervention types, and intervention focus on participants’ completion of cancer screening. Intervention effect sizes for participants’ completion of cancer screening were calculated using Hedge’s g statistic and were weighed by the sample size of the studies. The Hedge’s g-values were then averaged to calculate the overall effect size and converted to a z value. The Tau^2^ and I^2^ statistics were utilized to evaluate the included studies’ heterogeneity and reveal the variance among the studies. The I^2^ statistics values were categorized into no (0%–25%), low (25%–50%), moderate (50%–75%), and high (75%–100%) heterogeneity [31]. When necessary, raw data (e.g., mean with standard deviation) in the studies were converted to the other type of data (e.g., percentage). We assessed risk of publication bias within studies according to the PRISMA recommendation using a tool based on Agency for Healthcare Research and Quality’s guidance. Moreover, forest plots were prepared to visualize the effect size and the odds ratio with 95% CI. Publication bias was examined visually using funnel plots. An asymmetrical funnel plot represents a potential publication bias. The first author did the data analysis and the second author reviewed and verified the results.

## Results

### Search results

**Fig 1:**
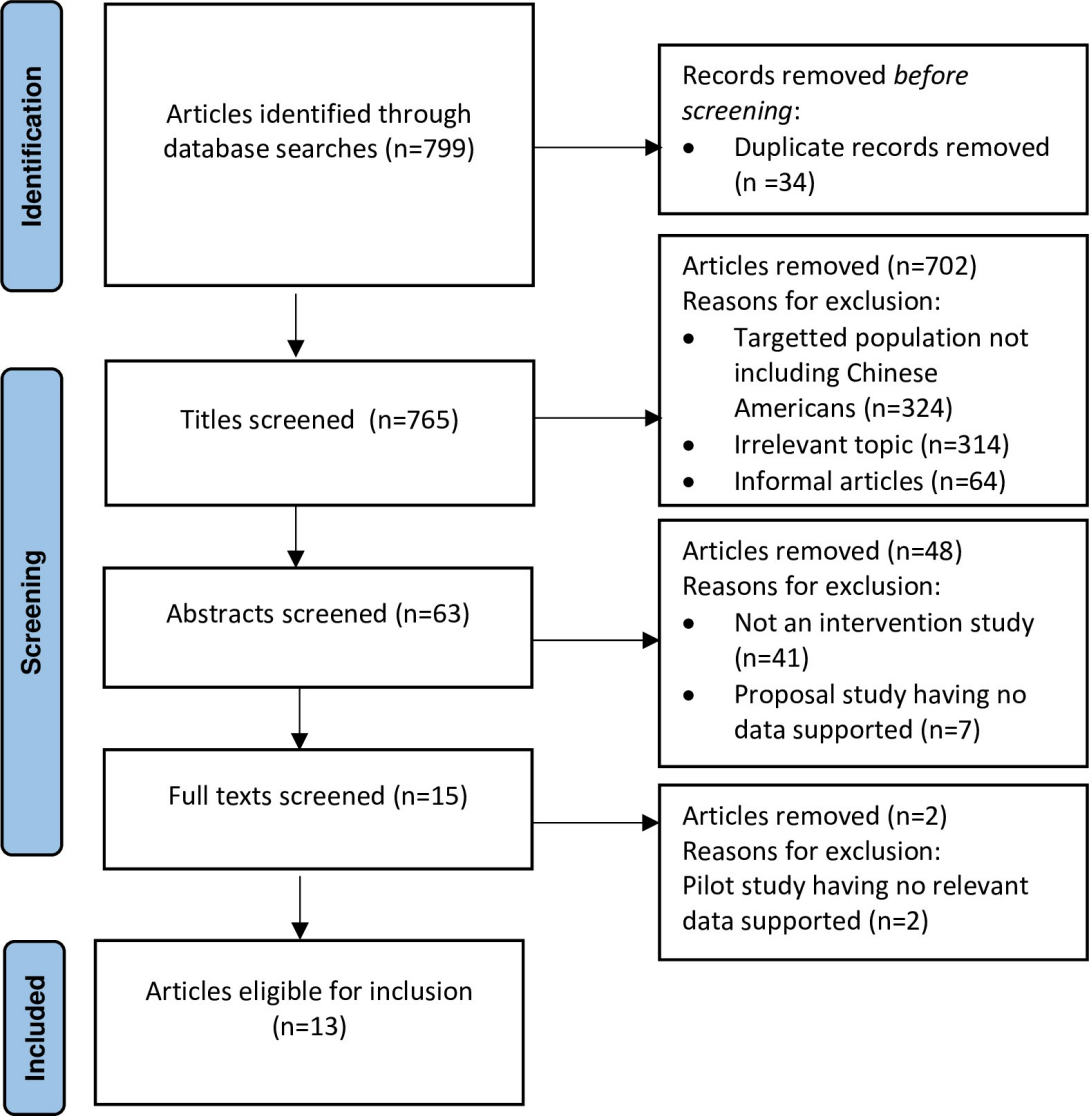
PRISMA flow chart documenting the study selection process.

Among the 799 articles found by the key-word searching and filtered by the publication date and full text, 702 and 48 articles were excluded per the exclusion criteria, in the process of inspecting the titles and abstracts of the articles, respectively; 34 articles were excluded due to replication and 2 articles were excluded in the full text inspection (Fig 1). The keyword searching process yielded 13 eligible articles [32–44] published from 2011 [32] to 2018 [33–35] (Table 1). Sample sizes of the studies ranged from 44 [36] to 3118 [44]. Four studies were quasi-experimental studies [32, 36–38], and nine were randomized control studies [33–35, 39–44]. Seven studies focused on the interventions on breast cancer screening with mammograms [32, 36, 38–42], five studies focused on the interventions on colorectal cancer screenings [34, 35, 37, 43, 44], and one study focused on the intervention on general cancer screening [33]. Specifically, results about the cultural and delivery characteristics of the interventions were systematically summarized, and the outcomes were meta-analyzed as shown below.

**Table 1:** Study characteristics of the included studies.

First author, date	Purpose	Study design	Sample, number of participants	Intervention		Patients’ outcome results	Quality score (PEDro score)
				Intervention group	Control group		
Maxwell et al., 2011 [32]	To evaluate the feasibility, acceptability and potential effect of a small-group video intervention led by trained Chinese American lay educators who recruited Chinese American women not up to date on mammography screening. • Type: Mammography screening	One group pre- and post-intervention study • Face-to-face in person educational seminar • Group-based intervention • Chinese American lay educators-led	Being Chinese American, over 40 years of age, not having had a mammogram during the past two years, and not having scheduled a mammogram in the next six months N = 101	A small-group video intervention led by trained Chinese American lay educators, a culturally tailored video promoting screening followed by a question-and-answer session and distribution of print materials. N = 101	NA	Screening completion/intent: 44% of the attendees reported receipt of a mammogram within 6 months after the small-group session with higher odds of screening among women who had lived in the U.S. less than 10% of their lifetime.	3
Fung Lei-Chun et al., 2018 [33]	To test the effect of an educational seminar on Cantonese-speaking Chinese Americans’ cancer screening intent. • Type: General cancer screening	Randomized controlled trail • Face-to-face in person educational seminar • Group-based intervention • Community health educators-led	Cantonese‐speaking Chinese Americans in San Francisco, age 18 years or older, able to attend a 2-hour session, fill out a questionnaire before and after the seminar, and willing to attend 1 of 2 seminars chosen by computer selection: “Cancer Screening and Prevention” (cancer prevention) or “Cancer Research in the Community” (biospecimen education). N = 395	Cancer prevention seminar Each seminar lasted 2 hours, including 1 hour for the seminar (PowerPoint presentation) and a total of 1 hour for a brief questionnaire administered before and after the seminars. The topics in the cancer prevention seminar included the following: 1) “What is cancer and who gets cancer?” (how cancer is the leading cause of death in Asian Americans); 2) “cancer myths and facts” (cancer is not contagious, and some cancers are curable); 3) “common cancers among Chinese men and women” (lung, colorectal, liver, stomach, and prostate cancers among men; lung, colorectal, breast, cervical, and thyroid cancers among women); 4) “risk factors for common cancers; 5) “early warning signs of common cancers”; 6) “American Cancer Society cancer screening guidelines” (for breast, cervical, colorectal, and prostate cancers); and 7) “general cancer risk reduction” (what individuals can do, including weight control, regular exercise, no smoking, drink alcohol in moderation, and using sun protection). N = 202	Biospecimen education seminar Biospecimen education (which increased willingness to donate biospecimens) N = 193	After the cancer prevention seminar, significant increases within group were noted for knowledge (eating healthy foods, from 93.1% to 97.7% [P = .0002]; second-hand smoke causes cancer, from 66.3% to 74.8% [P = .04]) and for screening completion/intent (colorectal cancer, from 58.1% to 64.5% [P = .002] cervical cancer, from 72.9% to 75.5% [P = .04]) and there was a trend toward an increase for prostate cancer (from 50.0% to 61.1%; P = .10). There was a significant change between groups for eating healthy foods (P = .004).	6
Sun et al., 2018 [34]	To assess the efficacy of an intervention initiated by a physician network that included Continuing Medical Education (CME) and mailed colorectal cancer (CRC) information and FOBT kit to increase CRC screening rates among Chinese Americans. Type: Colorectal cancer screening	Randomized controlled pilot trial • Individual-based intervention • Physicians-led	Chinese Americans, Current member of the Chinese Community Health Plan; between the ages of 50–75; had an estimated life expectancy of 10 years or more; and were not up to date on CRC screening (no FOBT within one year, sigmoidoscopy within five years, or colonoscopy within 10 years) from September 2006 through December 2009. N = 3118	The early intervention group primary care physicians (PCPs) received CME, and their patients received an intervention mailer, consisting of a letter with PCP’s recommendation, bilingual educational booklet, and FOBT kit in Year 1. N = 2020	The delayed intervention group PCPs received no CME, and their patients received the mailers in Year 2. N = 1098	FOBT screening rates increased from 26.7% at Baseline to 58.5% in Year 1 in the Early Intervention group vs. 19.6% to 22.2% in the Delayed Intervention group (p<. 0001). The overall effect size of the mailer intervention with or without CME was estimated as 26.6 percentage points (95% CI: 22.0–31.2) difference from Baseline compared to usual care.	6
Wang, Ma et al., 2018 [35]	To test the efficacy of an intervention to increase CRC screening by enhancing Chinese-speaking primary care physicians’ efficacy in communication about CRC screening to counteract Chinese American patients’ screening barriers and concerns. Type: Colorectal cancer screening	Cluster-randomized trial • Face-to-face intervention • Individual-based intervention • Physicians-led	Chinese Americans, 50–75 years old, active patients of participating physicians (visited within 2 years from the enrollment date), without a personal history of CRC, and non-adherent to the 2008 USPSTF CRC screening guidelines in place during the study period (including never screened, or last FOBT > 1 year, or sigmoidoscopy > 5 years, or colonoscopy > 10 years). N = 479	The intervention consisted of 3 components: a printed communication guide, 2 structured, in-office training sessions with simulated patients, and auxiliary materials, including a desk-style flip chart summarizing key points from the guide, FOBT instruction sheets for patients, and local free/low-cost screening information sheets. All materials were provided in both Chinese and English languages. Follow up time is 12 months after the intervention. N = 246	Physicians in the control arm practiced usual primary care and did not receive any intervention materials except the local free/low-cost screening information sheet. N = 233	Screening rates were slightly higher in the intervention vs. the control arm (24.4% vs. 17.7%, p = .24). In post hoc analyses, intervention arm patients who perceived better communication were more likely to be screened than those who did not (OR = 1.09, 95% CI: 1.03, 1.15). This relationship was not seen in the control arm.	6
Lee-Lin et al., 2013 [36]	To assess the feasibility and acceptability of a targeted educational intervention to increase mammography screening among Chinese American women. Type: Mammography screening	One-group pre- and post-test quasi-experimental design • Face-to-face in person educational seminar • Group-based intervention • Community-agencies-led	Being a foreign-born Chinese woman, being aged 40 years or older, having no history of breast cancer, being able to understand and read English or Chinese, not having had a mammogram within the past year, and having a phone and postal address. N = 44	A targeted breast health educational program Before starting the group session, participants completed a baseline survey, which was administered again 12 weeks postintervention. N = 44	NA	Of the 42 women who completed the study, 21 (50%) had a mammogram postintervention. Mean breast cancer susceptibility scores increased significantly at post-test as theorized (t [40] = –2.88, p < 0.01).	4
Wang, Burke et al., 2014 [37]	To explore the feasibility and acceptability of having traditional Chinese medicine (TCM) providers deliver education about CRC screening. Type: Colorectal cancer screening	One-group pre- and post-test quasi-experimental design • Face-to-face in person educational seminar • Group-based intervention • Physician-led	Self-identifying as Chinese and being aged 50 to 75, available for 2 meetings, and able to stay in the study for 3 months. N = 57	Four TCM providers (2 herbalists and 2 acupuncturists) were trained to deliver small-group educational sessions to promote CRC screening. Each provider recruited 15 participants. Participants completed a baseline survey on CRC-related knowledge, attitudes, and behaviors and then attended one 2-hour educational session delivered by the providers in Cantonese or Mandarin. Three months later, participants completed a postintervention survey. N = 57	NA	At post intervention, significant increases were found in having heard of CRC (from 52.6% to 79.0%, P < .001) and colon polyps (from 64.9% to 84.2%, P < .001). Knowledge regarding screening frequency recommendations also increased significantly. The rate of ever having received any CRC screening test increased from 71.9% to 82.5% (P < .001). The rate of up-to-date screening increased from 70.2% to 79.0% (P = .04).	4
Berger et al., 2017 [38]	To implement a three-phase peer-led community program designed to promote cancer prevention by improving breast cancer screening rates. Type: Mammography screening	One-group pre- and post-test quasi-experimental design • Face-to-face workshop and print materials • Group-based intervention • Community-based intervention	Chinese and Vietnamese women in the Greater Boston area N = 252	The workshop was one hour long and included a PowerPoint presentation with time for questions and answers, and handouts in English and Chinese, Komen shower cards, and Komen breast cancer stickers. It included 14 workshops and was implemented in 12 months. N = 252	NA	Results showed the majority of the women had received a clinical breast exam or mammogram in the past 12 months (69% and 59% respectively), and older women were more likely to get a mammogram (85%) or clinical breast exams (74%) compared to younger women. For questions with fewer correct answers at baseline, knowledge about the meaning of lumps in the breast significantly increased (69% to 80% correct, p<0.0001), as well as knowledge about frequency of clinical breast exam (48% to 67% correct, p<0.0001). Of the 192 participants who answered the question about willingness to get a mammogram, the majority (88%) were willing to receive one.	4
Sadler et al., 2012 [39]	This study hypothesizes that women who received information about breast cancer (breast cancer arm) will be more likely to adhere to current breast cancer screening guidelines between baseline and follow-up than those who received information about prostate cancer (prostate cancer arm). Type: Mammography screening	Randomized controlled trial • Face-to-face in person educational seminar • Group-based intervention • Student health educators-led	Women aged 40 and older N = 1,522, Chinese (n = 381); Filipino (n = 414); Korean (n = 371); and Vietnamese (N = 356).	Asian grocery store-based breast cancer education program Every woman in the breast cancer arm received the flyer describing the state’s free breast cancer screening program for low-income women. They were told how to access the program and to have an English speaker make the phone call, since only English and Spanish language lines were available. Along with this flyer, other information was given to expand the women’s knowledge of breast cancer, increase their motivation to become screened, and decrease barriers such as fear of the screening. Follow up time is 8 weeks after intervention. N = 813	Prostate cancer education program The prostate cancer education program arm received an equivalent intervention for prostate cancer. N = 709	Women aged 40 and older and non-adherent for annual screening mammograms were more likely to schedule a mammogram after receiving the breast cancer education program than women randomized to the prostate cancer program (X^2^ = 3.85, p = 0.05).	5
Wu et al., 2015 [40]	To develop and test a tailored intervention for Asian American women regarding the breast cancer screening. Type: Mammography screening	A randomized control single blind study • Telephone-based intervention • Individual-based intervention • Community educator-led	Self-identification as either Chinese or Taiwanese Americans, are age 41 and older; not had a mammogram within the past 15 months; never been diagnosed with breast cancer; and can read and speak English or Chinese. N = 193	Individually tailored telephone counseling The intervention group members received an intervention tailored to the results of their baseline interviews. For example, women with responses of “agree or strongly agree” on barriers items, “disagree or strongly disagree” on benefits and self-efficacy items, or incorrectly answered knowledge items were provided with counseling messages related to those items. Self-reported data that included demographic variables, knowledge, beliefs, and screening behaviors were collected at baseline and 4 months. N = 96	National Cancer Institute brochure The control group received a mammography pamphlet on breast health developed by the (NCI). The NCI brochure explains the procedure of mammography and the importance of early detection through mammography. N = 97	The intervention group had increased screening to 40% compared with 33% for the control group at 4 months; the difference was not statistically significant.	5
Wang, Schwartz, Brwon et al., 2012 [41]	To examine the efficacy of the cultural and generic videos in increasing Chinese American immigrant women’s mammography screening behavior relative to a control group that received a fact sheet. Type: Mammography screening	Three-arm randomized controlled trial • Individual-based intervention • Community-based intervention	Self-identified as Chinese American; were over the age of 40; lived in the Washington, DC or New York City metropolitan areas; had no personal history of breast cancer; were non-adherent to the American Cancer Society annual mammography screening guideline and had no medical appointment for a mammogram within the six months following the enrollment period. N = 571	A culturally targeted video, a generic video Trained bilingual interviewers utilized a computer assisted telephone interview (CATI) system to conduct baseline and two follow-up assessments. Participants were randomized immediately after they completed baseline assessment. Intervention materials were mailed to participants’ homes within a week after randomization. Two to four weeks after materials were mailed, participants were called to confirm receipt and review of the materials. At that time, the first follow-up survey was administered to collect feedback on the materials and again measure key variables of knowledge, Eastern cultural views of healthcare, and health beliefs since baseline (for process evaluation). Women who had not yet reviewed the materials were asked to do so before the follow-up interview. Results from the process evaluation indicated that all participants were able to recall content from key sections of the materials. The second follow-up assessment (outcome evaluation) was administered six months post intervention to measure mammography screening behavior. All participants were interviewed in Chinese (Mandarin and Cantonese) languages. N = 191, N = 187	A fact sheets N = 193	The culturally targeted video, the generic video, and the fact sheet increased mammography utilization by 40.3%, 38.5%, and 31.1% from baseline, respectively.	6
Wang, Schwartz, Luta et al., 2012 [42]	To compare a culturally tailored video promoting positive attitudes toward mammography among Chinese immigrant women to a linguistically appropriate generic video and print media. Type: Mammography screening	Randomized controlled trial • Individual-based intervention • Community-based intervention	Chinese American women over the age of 40, immigrants from the metropolitan Washington, DC, and New York City areas, with no personal history of breast cancer, who had not adhered to the American Cancer Society annual mammography screening guidelines and had not already scheduled an appointment for a mammogram within the 6 months following the enrollment period. N = 592	Culturally tailored video, A linguistically appropriate generic video Videos were mailed to the participants. The cultural video had the following features: (i) an all-Chinese cast, (ii) a soap opera set within the lives of a Chinese family, (iii) Chinese dialog featuring appropriate idioms, (iv) Chinese foods and decor at the birthday party and (v) Chinese background music. The generic video targets common barriers to mammography use such as lack of knowledge, fear of pain and radiation, concerns about cost and time, fatalistic beliefs, and low perceived risk for breast cancer. Beeline and post-intervention questionnaires were conducted on telephone. Follow up time was 2–4 weeks after intervention. N = 198, N = 195	Print media N = 199	Results showed that both videos improved screening knowledge, modified Eastern views of health care, reduced perceived barriers and increased screening intentions relative to print media (all P < 0.05). The generic video increased screening intention twice as much as the cultural video, although subgroup analysis showed the increase was only significant in women aged 50–64 years. Only Eastern views of health care were negatively associated with screening intentions after adjusting for all baseline covariates.	5
Carney et al., 2014 [43]	To test the impact of an educational intervention delivered by specially trained community health workers among Chinese, Korean, and Vietnamese participants aged 50–75 on knowledge, attitudes, beliefs, and intention regarding colorectal cancer screening. Type: Colorectal cancer screening	A randomized controlled trial • Face-to-face workshop • Group-based intervention • Community health workers-led	Men and women of Chinese, Vietnamese or Korean heritage aged 50–75 years, with no prior history of receiving CRC screening within the past 5 years, no prior personal or family history of colon cancer, no major medical illnesses that would preclude them from receiving CRC screening, the ability to sign informed consent, and willingness to be randomly assigned to one two groups and participate in educational intervention. Those with a first-degree relative with colon cancer, other household members of an enrolled participant, and those with significant medical problems were excluded. N = 654	An educational intervention delivered by specially trained community health workers A community health worker provided background information, which was collaboratively designed by the community and the research team, on the importance of colorectal cancer screening, what tests are available for this, where to go to obtain the tests and where to go to get the tests for free or at a reduced cost. If patients identified themselves as needing a primary care provider, they were assisted in finding one by staff in the Asian Health and Service Center. Health messages that help overcome barriers people experience when thinking about getting colorectal cancer screening was provided to the participants in a way that allowed them to be active learners. PowerPoint presentation was used. Questions and answer sessions were also held. It included 15 sessions. N = 329	Educational pamphlets in their native language All participants received American Cancer Society educational brochures about colorectal cancer screening in English and in their primary language. N = 325	Results showed the changes on perceived Behavior Control and Intentions (pre- vs. post- change in control group −0.16; change in intervention group 0.11, p = 0.004), Behavioral Beliefs on Cancer Screening (pre- vs. post- change in control group −0.06; change in intervention group 0.24, p = 0.0001), and for Attitudes Toward Behavior (pre- vs. post- change in control group −0.24; change in intervention group 0.35, p = <0.0001). The intervention had no effect on Behavioral Beliefs on Cancer, Control Beliefs, and Perceived Behavioral Control (Reliance on Family). Though intention to stay up to date for cancer screening increased in two study groups (Chinese and Vietnamese), these were not significant.	5
Nguyen et al., 2017 [44]	To compare the efficacy of two interventions in increasing CRC screening among Chinese Americans. Type: Colorectal cancer screening	Cluster randomized comparative trial • Face-to-face in person group sessions and telephone call intervention + print materials • Group and individual intervention • Lay Health worker-led	Age 50–75 years; self-identifying as Chinese American; speaking English, Cantonese, or Mandarin; residing in San Francisco with intention to stay for 6 months; no personal history of CRC; and no other participants in the same household. N = 725	Lay health worker (LHW) intervention plus in-language brochure (LHW+Print). LHWs in the LHW+Print arm were trained to teach participants about CRC in two small group sessions and two telephone calls. Follow up time is 6-months post-intervention. N = 360	Brochure (Print) N = 365	Knowledge increase was significant (p<0.002) for nine measures in the LHW+Print group and six in the Print group. Both groups had increases in having ever been screened for CRC (LHW+Print, 73.9% to 88.3%, p<0.0001; Print, 72.3% to 79.5%, p = 0.0003) and being up to date for CRC screening (LHW+Print, 60.0% to 78.1%, p<0.0001; Print, 58.1% to 64.1%, p = 0.0003).	5

### Study quality

Of the 13 eligible papers, four were good quality trials [33–35, 41], nine were fair quality trials [36–40, 42–44], and one was a poor-quality trial [32]. The score of each individual study’s quality constituted the average value of the scores given by the two assessors. It ranged from 3 to 6 points (mean = 5) (Tables 1 and 2). The ICC was 0.88 (95% CI: 0.47–0.99).

**Table 2:** Methodological quality measurement of included studies (PEDro scale).

PEDro variables	No. Studies	References
Random allocation	9	[33–35, 39–44]
Concealed allocation	4	[33–35, 39]
Baseline comparability	13	[32–44]
Blinding of participants	0	0
Blinding of therapists	0	0
Blinding of assessors	0	0
Adequate follow-up (> 85%)	11	[33, 34, 36–38, 40–44]
Intention-to-treat analysis	2	[35, 41]
Between-group statistical comparisons	13	[32–44]
Reporting of point measures and measures of variability	13	[32–44]

### Descriptions of interventions based on the systematic review

#### Cultural characteristics of the interventions

For the interventions which were conducted in the 13 studies, all of them were culturally tailored interventions which were delivered in the Mandarin and/or Cantonese spoken language, or Chinese written language. Cultural characteristics such as Chinese beliefs (e.g., fatalistic views of cancer, yin-yang balance in the body, attitudes toward Western examinations, embarrassment towards diseases), social and family support, and language barriers were considered when designing the group-based interventions [42, 43]. Culturally adapted materials for the individual-based interventions were provided in both Chinese and English. Several Chinese culture elements were reflected in the videos for the individual-based interventions [41, 42]. For example, they used an all-Chinese cast, a soap opera set within the lives of a Chinese family, Chinese dialog featuring appropriate idioms, Chinese foods and decorations at the settings, and Chinese background music [42].

#### Delivery characteristics of the interventions

We organized the characteristics of the intervention delivery methods and outcomes into logical categories according to group consensus [45]. The primary comparator was usual care for the randomized control trails and the pre-intervention for the pre-post intervention studies. For trials with multiple arms, we assessed the outcomes of the culturally tailored interventions compared to usual care. The characteristics about the delivery methods and outcomes related to the interventions are summarized in Table 3.

**Table 3:** Intervention characteristics of the included studies.

Citation	Intervention methods	Intervention delivery objects	Intervention led	Intervention contact	Intervention types	Intervention focus
Maxwell et al., 2011 [32]	Small-group video intervention + a question-and-answer session + distributed a Chinese pamphlet + a list of local facilities providing low- or no-cost screening mammograms	group	community worker or educator	in-person	patient education	client-focused
Fung Lei-Chun et al., 2018 [33]	PowerPoint presentation cancer prevention seminar	group	community worker or educator	in-person	patient education	client-focused
Sun et al., 2018 [34]	PCPs received Continuing Medical Education (CME); Their patients received an intervention mailer (a letter with PCP’s recommendation + bilingual educational booklet + FOBT kit)	individual	physician	in-person	clinician education + Patient education + screening kit outreach	client and clinician-focused
Wang, Ma et al., 2018 [35]	PCPs received a communication guide and 2 in-office training sessions on communicating CRC screening with patients	individual	physician	in-person	clinician education	Clinician-focused
Lee-Lin et al., 2013 [36]	A targeted breast health educational program: an hour-long class + individual counseling sessions by phone to help participants overcome barriers	group	community worker or educator	in-person	patient education + patient navigator	client-focused
Wang, Burke et al., 2014 [37]	Four TCM providers were trained to deliver small-group educational sessions; Their patients received one 2-hour educational session delivered by the providers about CRC prevention using the flipchart, followed by a group discussion	group	physician	in-person	clinician education + Patient education	Client and clinician-focused
Berger et al., 2017 [38]	Fourteen workshops included a PowerPoint presentation with time for questions and answers + handouts, Komen shower cards + Komen breast cancer stickers	group	community worker or educator	in-person	patient education	client-focused
Sadler et al., 2012 [39]	Asian grocery store-based breast cancer education program: brief face-to-face education session + flyer describing the state’s free breast cancer screening program for low income women + information about how to access the program and have an English speaker make the phone call for them + other information about knowledge of breast cancer and decrease barriers	individual	community worker or educator	in-person	patient education	client-focused
Wu et al., 2015 [40]	A Web-based, individually tailored program for the telephone counseling component which tailored to the results of their baseline interviews	individual	community worker or educator	indirect remote	patient navigator	client-focused
Wang, Schwartz, Brwon et al., 2012 [41]	Mailed intervention videos: culturally targetted video, a generic video, and a fact sheet (control)	individual	community worker or educator	indirect remote	patient education	client-focused
Wang, Schwartz, Luta et al., 2012 [42]	Mailed intervention videos: culturally targetted video, a generic video, and a fact sheet (control)	individual	community worker or educator	indirect remote	patient education	client-focused
Carney et al., 2014 [43]	Fifteen intervention sessions, health education information + assisted in finding one primary care provider if needed + health messages that help overcome barriers	group	community worker or educator	in-person	patient education + patient navigator	client-focused
Nguyen et al., 2017 [44]	Lay health worker (LHW) intervention + in-language brochure vs brochure. LHWs in the LHW+Print arm were trained to teach participants about CRC in two small group sessions and two telephone calls.	group	community worker or educator	in-person	patient education	client-focused

*Intervention delivery objects*. In the studies, a variety of intervention delivery methods were noted. Six studies were individual-based intervention studies [34, 35, 39–42], and seven studies were group-based intervention studies [32, 33, 36–38, 43, 44]. The individual-based interventions were conducted using culturally adapted mailed information packages [34], mailed videos [41, 42], in-person consultations [35, 39], or individually tailored telephone counseling [40]. The group-based interventions were held in churches, community-based organizations/offices, private residences, hospitals, senior centers, or physicians’ offices [32–44]. Durations for the group-based workshops ranged from 60 minutes [36] to 120 minutes [38]. Each group session was held with 5 to 8 attendees per group [37]. Question and answer sessions; Chinese language pamphlets, brochures, information sheets [32, 43, 44]; group discussions; flipcharts [37, 44]; or follow-up individual telephone counseling [36] were provided in workshops.

*Intervention led*. Three studies were physician-led intervention studies [34, 35, 37] and ten were community worker or educator-led studies [32, 33, 36, 38–44]. Two of the three physician-led intervention studies included two components, which were the physician-targeted components and patient-targeted components [34, 37]. The last physician-led intervention study had only one physician-targeted component, which aimed to indirectly increase the uptake rate of cancer screening among their patients [35]. In the three physician-led studies, physicians received trainings or seminars, or information materials related to screenings [34, 35, 37], and their patients received mailers [34] or small group sessions [37].

*Intervention contact*. Ten studies were direct in-person face-to-face intervention studies [32–39, 43, 44] and three were indirect remote or self-learning intervention studies [40–42]. The direct in-person face-to-face interventions were conducted either through in-person group workshops/sessions [32, 33, 36–38, 43, 44], through visits with physicians [34, 35, 37], or with community educators in the booths located in the Asian stores [39]. The indirect remote or self-learning interventions were conducted either by individually tailored telephone counseling [40] or mailed videos [41, 42].

*Intervention types*. In the studies, four types of interventions were identified, including patient education, clinician education, screening kit outreach, and patient navigator (a barriers-focused intervention). Among the 13 studies, nine studies used single component interventions, including seven studies which only used the patient education method [32, 33, 38, 39, 41, 42, 44], and two studies used the patient navigator method [40] and clinician education method [35], respectively; the other four studies [34, 36, 37, 43] used multiple-component interventions which included two or three components of the four intervention types.

*Intervention focus*. Three types of interventions focus were identified in the studies, including client-focused, clinician-focused, and both client and clinician-focused. Among the 13 studies, 10 studies used the client-focused method [32, 33, 36, 38–44], and their interventions focused on the clients; one study used clinician-focused method [35]; and two studies focused both on the patients and clinicians [34, 37].

### Intervention outcomes based on the meta-analysis

To measure outcomes of the interventions, nine studies tested effects of interventions on participants’ knowledge of cancer screening [32, 33, 36–38, 41–44]; seven studies tested effects of interventions on participants’ beliefs toward cancer screening [32, 33, 36, 37, 41–43]; four studies tested effects of interventions on participants’ attitudes toward cancer screening [32, 33, 37, 43]; eight studies tested effects of interventions on participants’ intentions to complete cancer screening [32, 33, 35, 37, 38, 42–44]; and ten studies tested effects of interventions on participants’ completions of cancer screening [33–41, 44].

Due to a vague and inconsistent definition of beliefs and attitudes in the available studies which tested effect of interventions on participants’ attitudes and beliefs toward cancer screening, which could bring possible bias to the results, this study did not conduct further meta-analysis exploring effects of interventions on participants’ attitudes and beliefs toward cancer screening. Only effects of interventions on participants’ knowledge of cancer screening, intentions to complete cancer screening, and completion of cancer screening were analyzed.

#### Effect on participants’ knowledge of cancer screening

**Fig 2:**
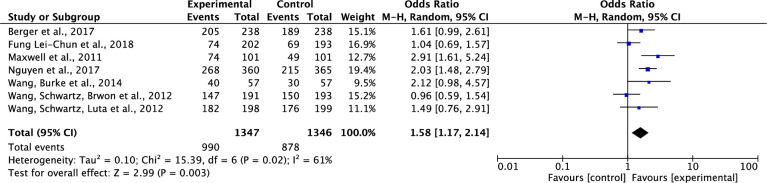
Forest plot of participants’ knowledge of cancer screening.

Of the nine studies which tested the effects of interventions on participants’ knowledge of cancer screening [32, 33, 36–38, 41–44], two studies were not included in the meta-analysis due to missing data on the total points which were used to measure knowledge level [36] and a vague measurement of knowledge in the report [43]. Results showed that compared to the control group, the group that received interventions on cancer screening had a significantly increased knowledge on cancer screening at post-intervention. The pooled summary effect of the interventions included was about one and a half times higher in comparison to the control (OR, 1.58; 95% CI, 1.17–2.14; P = 0.003). However, a moderate level of heterogeneity was noticed across the study results (Tau^2^ = 0.1, ChI^2^ = 15.39, df = 6, p = 0.02, I^2^ = 61%) (Fig 2).

#### Effect on participants’ intention to complete cancer screening

**Fig 3:**
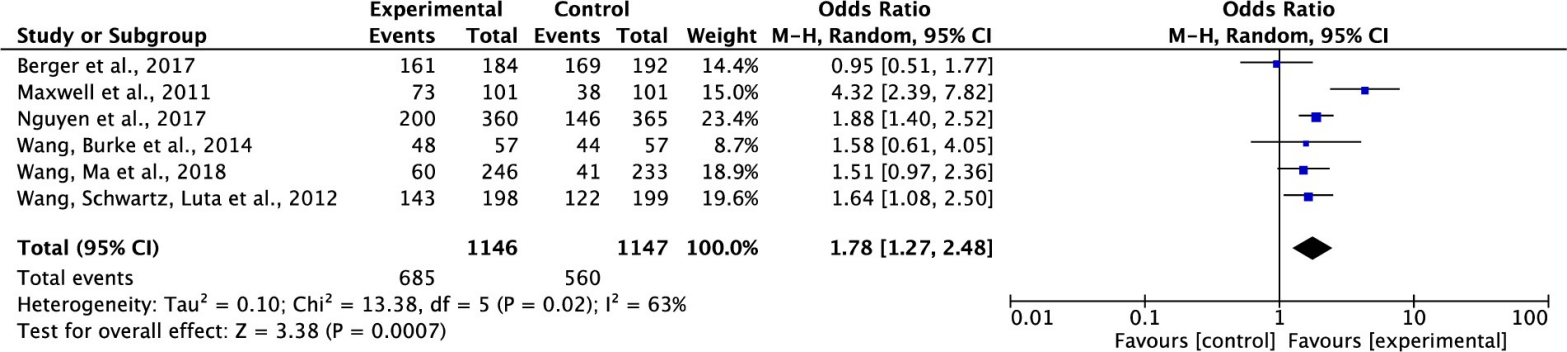
Forest plot of participants’ Intention to complete cancer screening.

Eight studies tested effects of interventions on participants’ intentions to complete cancer screening [32, 33, 35, 37, 38, 42–44]. Two studies were not included in the data analysis, because one study included participants’ completions of cancer screening data and intentions to complete cancer screening data together [33], the other study had missing data of the total points which were used to measure participants’ intentions to complete cancer screening [43]. Results showed that compared to the control group, the interventions on cancer screening significantly increased participants’ intentions to complete cancer screening. The pooled summary effect of the interventions included was about 1.78 times higher in comparison to the control (OR, 1.78; 95% CI, 1.27–2.48; P = 0.0007). Also, a moderate level of heterogeneity was noticed across these study results (Tau^2^ = 0.10, ChI^2^ = 13.38, df = 5, p = 0.02, I^2^ = 63%) (Fig 3).

#### Effect on participants’ completion of cancer screening

**Fig 4:**
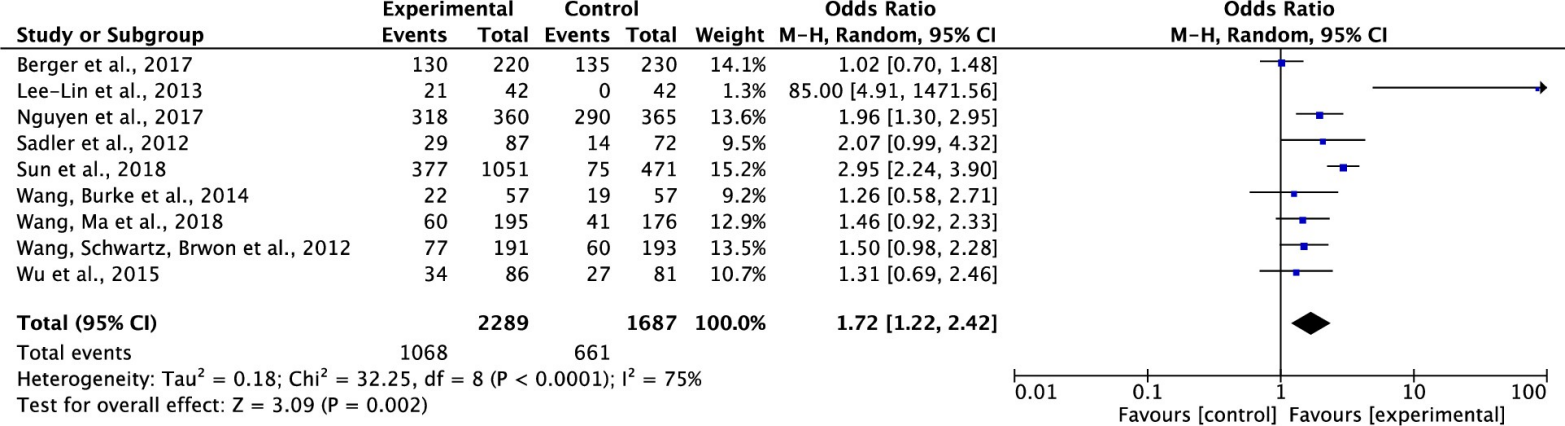
Forest plot of participants’ completion of cancer screening.

Of the ten studies testing effects of interventions on participants’ completions of cancer screening [33–41, 44], one study was not included in the data analysis, because it included participants’ completions of cancer screening data and intentions to complete cancer screening data together [33]. Results showed that compared to the control group, the interventions on cancer screening significantly increased participants’ completions of cancer screening. The pooled summary effect of the interventions included was about 1.72 times higher in comparison to the control group (OR, 1.72; 95% CI, 1.22–2.42; P = 0.002). Nevertheless, these results should be interpreted with caution due to the presence of a high level of heterogeneity (Tau^2^ = 0.18, ChI^2^ = 32.25, df = 8, p<0.0001, I^2^ = 75%) (Fig 4).

#### Subgroup analysis

**Fig 5:**
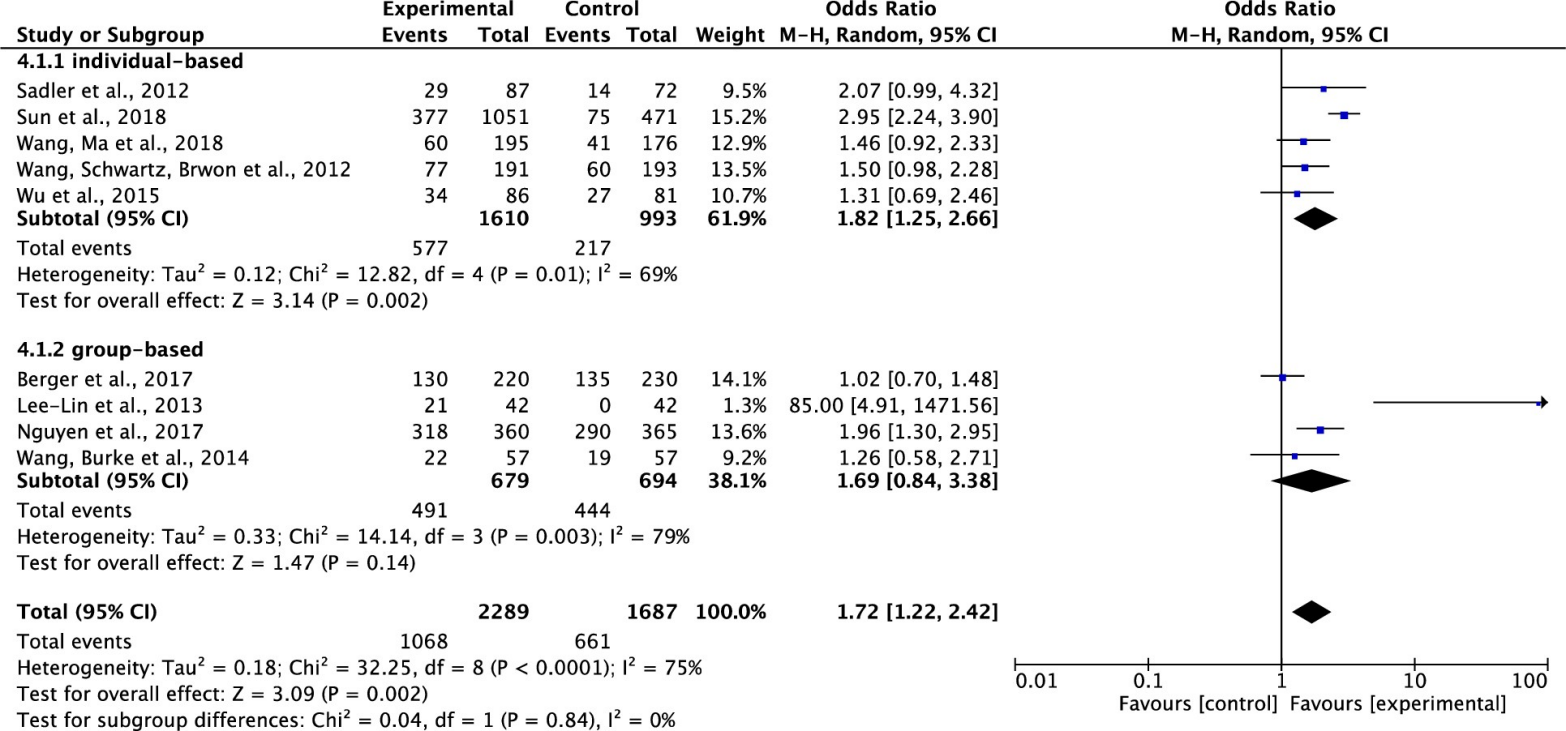
Forest plot of individual- VS. group-based interventions on participants’ completion of cancer screening.

*Comparison of the effects of individual- VS*. *group-based interventions on participants’ completion of cancer screening*. Of the nine included studies which tested effects of interventions on participants’ completions of cancer screening [34–37, 38–41, 44], five studies were individual-based intervention studies [34, 35, 39–41], and four studies were group-based intervention studies [36–38, 44]. Results showed that compared to the control group, the individual-based interventions on cancer screening significantly increased participants’ completions of cancer screening. The pooled summary effect of the individual-based interventions included was about 1.82 times higher, compared to the control (OR, 1.82; 95% CI, 1.25–2.66; P = 0.002); the same effect was noticed on the group-based interventions; however, the increase was not significant (OR, 1.69; 95% CI, 0.84–3.38; P = 0.14). Although with subgroup analysis, the heterogeneity across the studies decreased among individual-based studies (Tau^2^ = 0.12, ChI^2^ = 12.82, df = 4, p = 0.01, I^2^ = 69%), the total heterogeneity was high across all the studies (Tau^2^ = 0.18, ChI^2^ = 32.25, df = 8, p<0.0001, I^2^ = 75%) (Fig 5).

**Fig 6:**
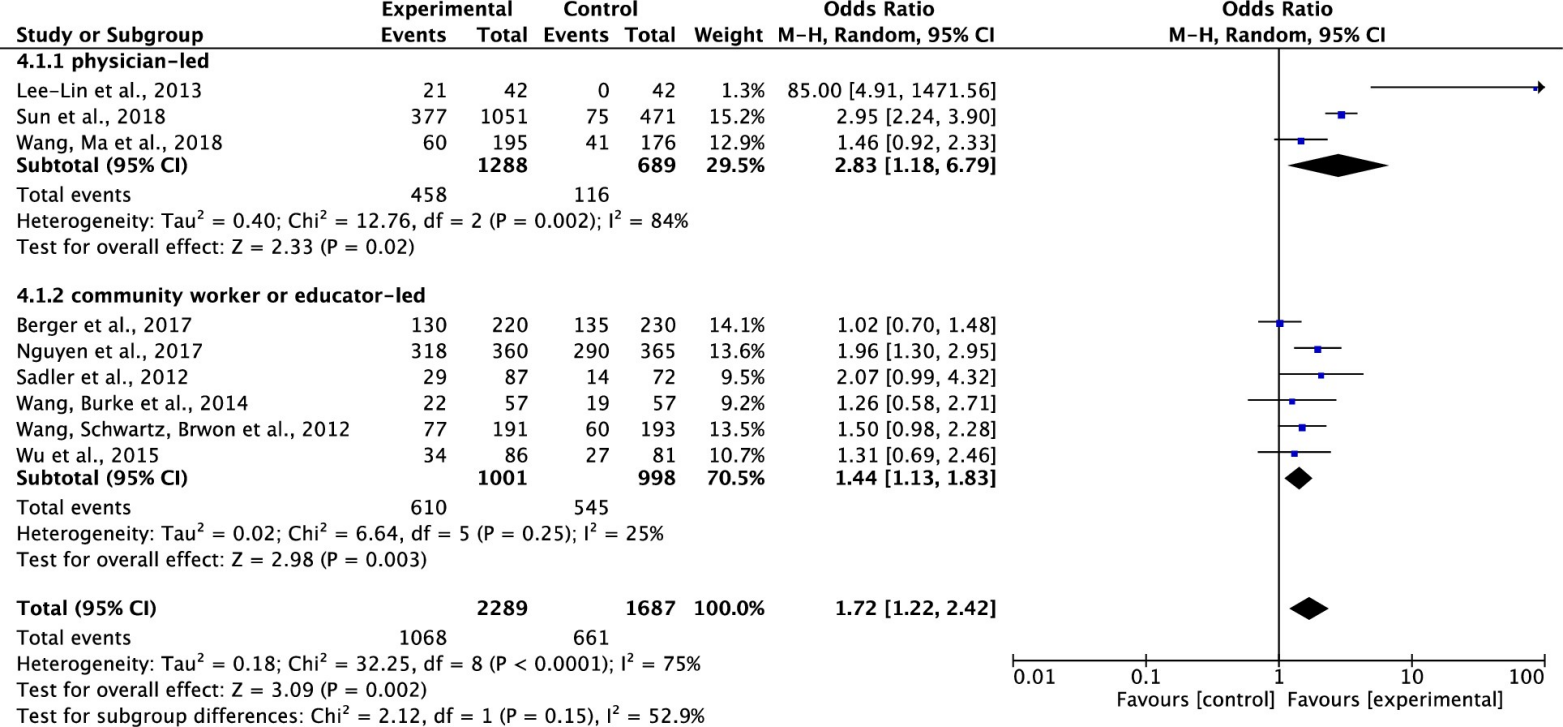
Forest plot of physician- VS. community worker or educator-led interventions on participants’ completion of cancer screening.

*Comparison of the effects of physician- VS*. *community worker or educator-led interventions on participants’ completion of cancer screening*. Of the nine included studies which tested effects of interventions on participants’ completions of cancer screening [34–37, 38–41, 44], three studies were physician-led intervention studies [34, 35, 37] and six were community worker or educator-led studies [36, 38–41, 44]. Results showed that compared to the control group, both the physician-led and the community worker or educator-led interventions on cancer screening significantly increased participants’ completions of cancer screening. The pooled summary effects of the physician-led and the community worker or educator-led interventions were about 2.83 times, and 1.44 times higher in comparison to the control, respectively (OR, 2.83; 95%CI, 1.18–6.79; P = 0.02 and OR, 1.44; 95% CI, 1.13–1.83; P = 0.003). Although the total heterogeneity was high across the studies (Tau^2^ = 0.18, ChI^2^ = 32.25, df = 8, p<0.0001, I^2^ = 75%), the heterogeneity across the studies significantly decreased in the subgroup analysis on community worker or educator-led studies (Tau^2^ = 0.02, ChI^2^ = 6.64, df = 5, p = 0.25, I^2^ = 25%) (Fig 6).

**Fig 7:**
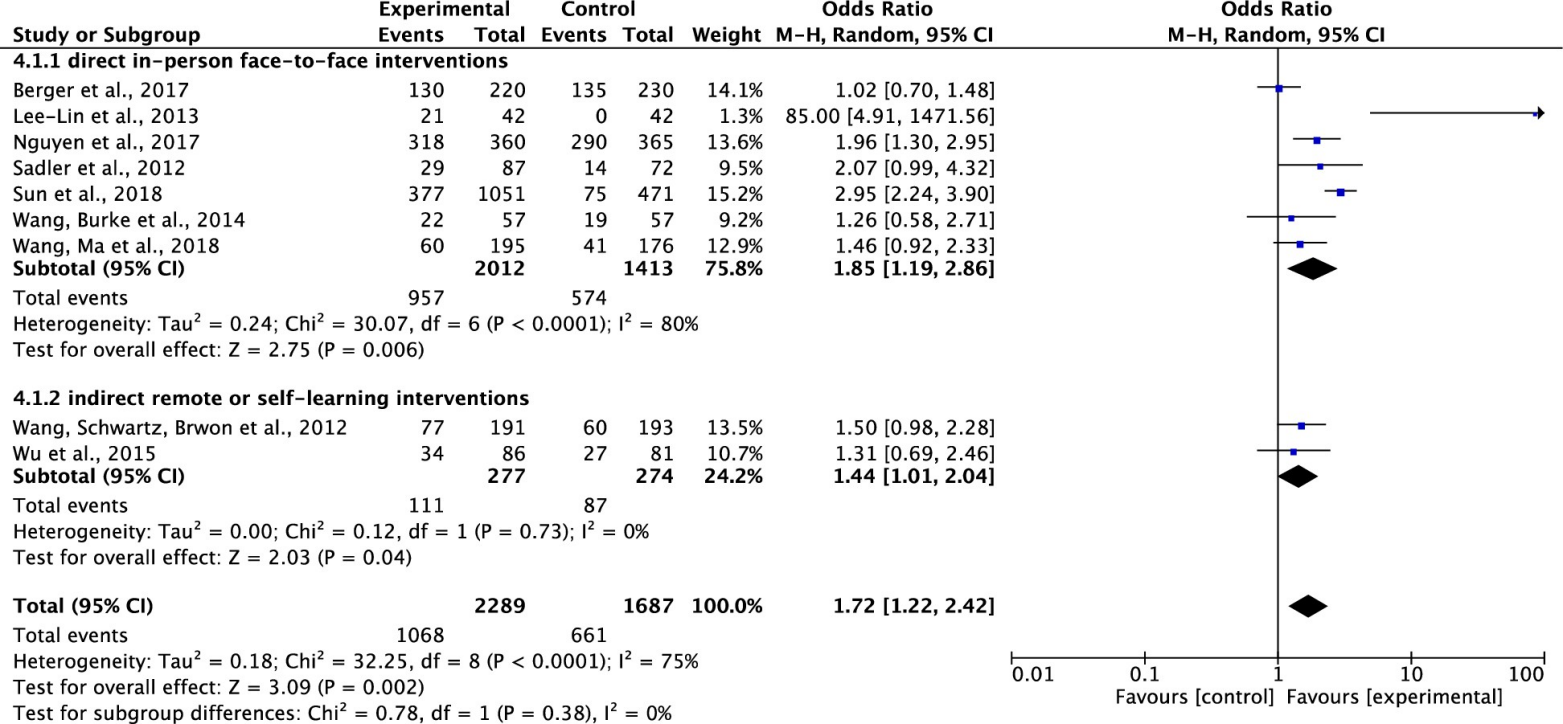
Forest plot of direct in-person face-to-face VS. indirect remote or self-learning interventions on participants’ completion of cancer screening.

*Comparison of the effects of direct in-person face-to-face VS*. *indirect remote or self-learning interventions on participants’ completion of cancer screening*. Of the nine included studies which tested effects of interventions on participants’ completions of cancer screening [34–41, 44], seven studies were direct in-person face-to-face intervention studies [34–39, 44] and two were indirect remote or self-learning intervention studies [40, 41]. Results showed that compared to the control group, both the direct in-person face-to-face interventions and the indirect remote or self-learning interventions on cancer screening significantly increased participants’ completions of cancer screening. The pooled summary effects of the interventions were about 1.85 times and 1.44 times higher in comparison to the control (OR, 1.85; 95%CI, 1.19–2.86; P = 0.006 and OR, 1.44; 95% CI, 1.01–2.04; P = 0.04, respectively). Although the total heterogeneity was high across the studies (Tau^2^ = 0.18, ChI^2^ = 32.25, df = 8, p<0.0001, I^2^ = 75%), absence of heterogeneity was noticed in the subgroup analysis on indirect remote or self-learning intervention studies (Tau^2^ = 0.00, ChI^2^ = 0.12, df = 1, p = 0.73, I^2^ = 0%) (Fig 7).

**Fig 8:**
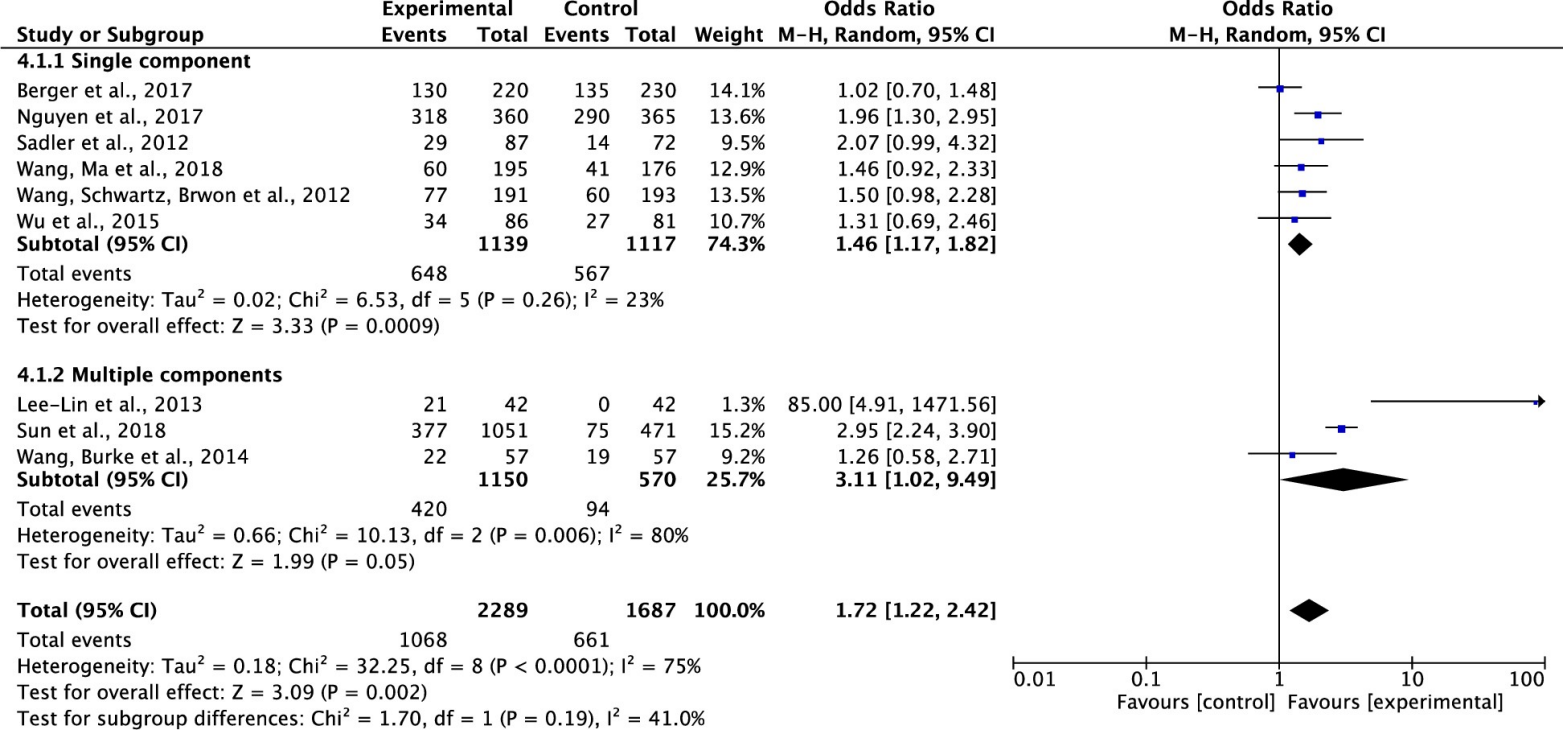
Forest plot of single component VS. multiple-component interventions on participants’ completion of cancer screening.

*Comparison of the effects of single component VS*. *multiple-component interventions on participants’ completion of cancer screening*. Of the nine included studies which tested effects of interventions on participants’ completions of cancer screening [34–41, 44], six studies were single component intervention studies [35, 38–41, 44] and three were multiple-component intervention studies [34, 36, 37]. Results showed that compared to the control group, both the single and multiple-component interventions on cancer screening significantly increased participants’ completion of cancer screening. The pooled summary effects of the single component and multiple-component interventions were about 1.46 and 3.11 times higher in comparison to the control (OR, 1.46; 95%CI, 1.17–1.82; P = 0.009 and OR, 3.11; 95% CI, 1.02–9.49; P = 0.05, respectively). Although the total heterogeneity was high across the studies (Tau^2^ = 0.18, ChI^2^ = 32.25, df = 8, p<0.0001, I^2^ = 75%), a decrease of heterogeneity was noticed in the subgroup analysis on single component intervention studies (Tau^2^ = 0.02, ChI^2^ = 6.53, df = 5, p = 0.26, I^2^ = 23%) (Fig 8).

**Fig 9:**
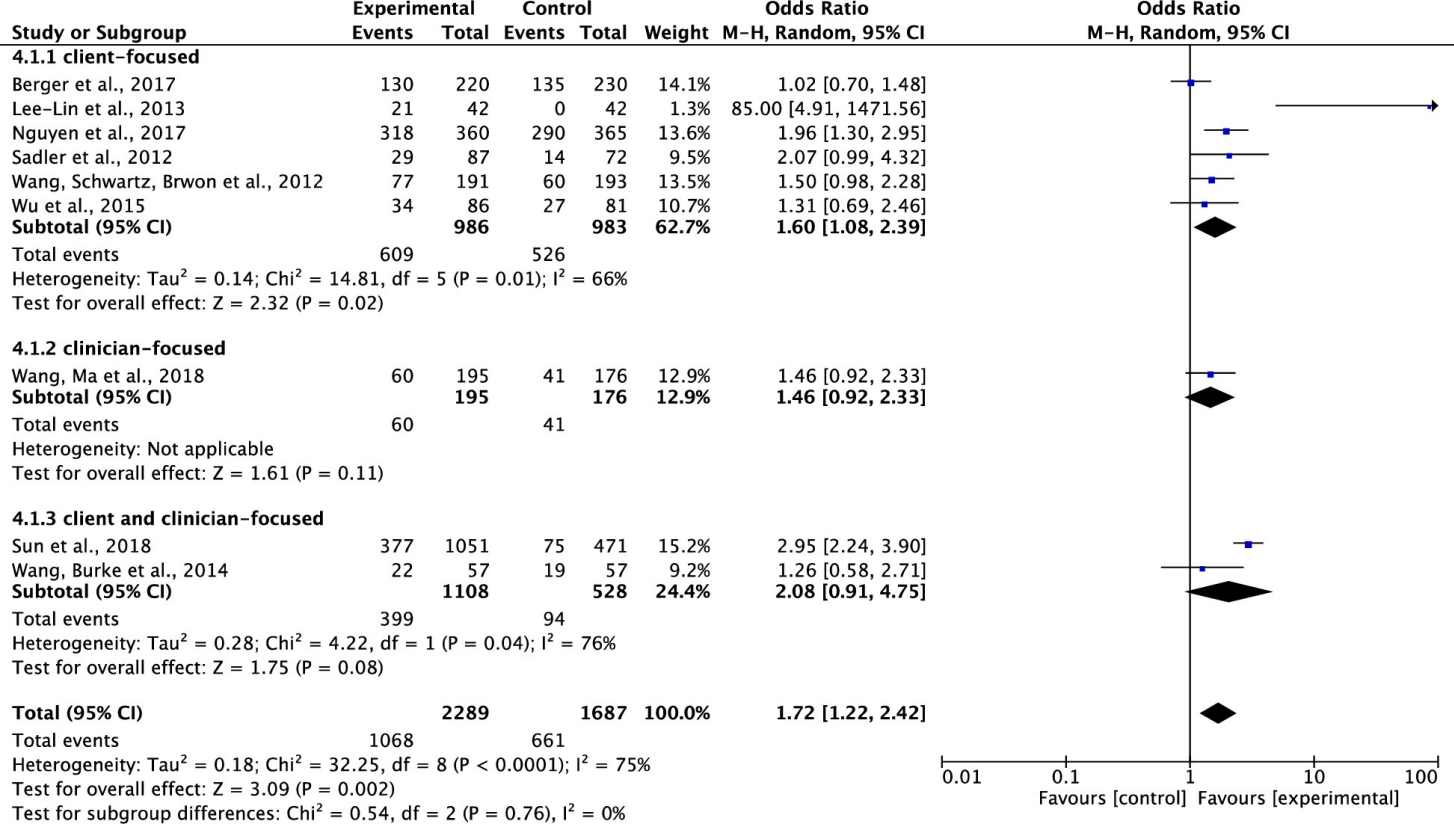
Forest plot of client-focused VS. clinician-focused VS. client and clinician-focused interventions on participants’ completion of cancer screening.

*Comparison of the effects of client-focused VS*. *clinician-focused VS*. *client and clinician-focused interventions on participants’ completion of cancer screening*. Of the nine included studies which tested effects of interventions on participants’ completions of cancer screening [34–41, 44], six studies were client-focused intervention studies [36, 38–41, 44], one was clinician-focused intervention study [35], and two were client and clinician-focused studies [34, 37]. Results showed that compared to the control group, the client-focused intervention on cancer screening significantly increased participants’ completion of cancer screening. The pooled summary effect of the client-focused intervention was about 1.6 times higher, compared to the control (OR, 1.60; 95%CI, 1.08–2.39; P = 0.02). Although the total heterogeneity was high across the studies (Tau^2^ = 0.18, ChI^2^ = 32.25, df = 8, p<0.0001, I^2^ = 75%), a decrease of heterogeneity was noticed in the subgroup analysis on client-focused intervention studies (Tau^2^ = 0.14, ChI^2^ = 14.81, df = 5, p = 0.01, I^2^ = 66%) (Fig 9).

### Publication bias

**Fig 10:**
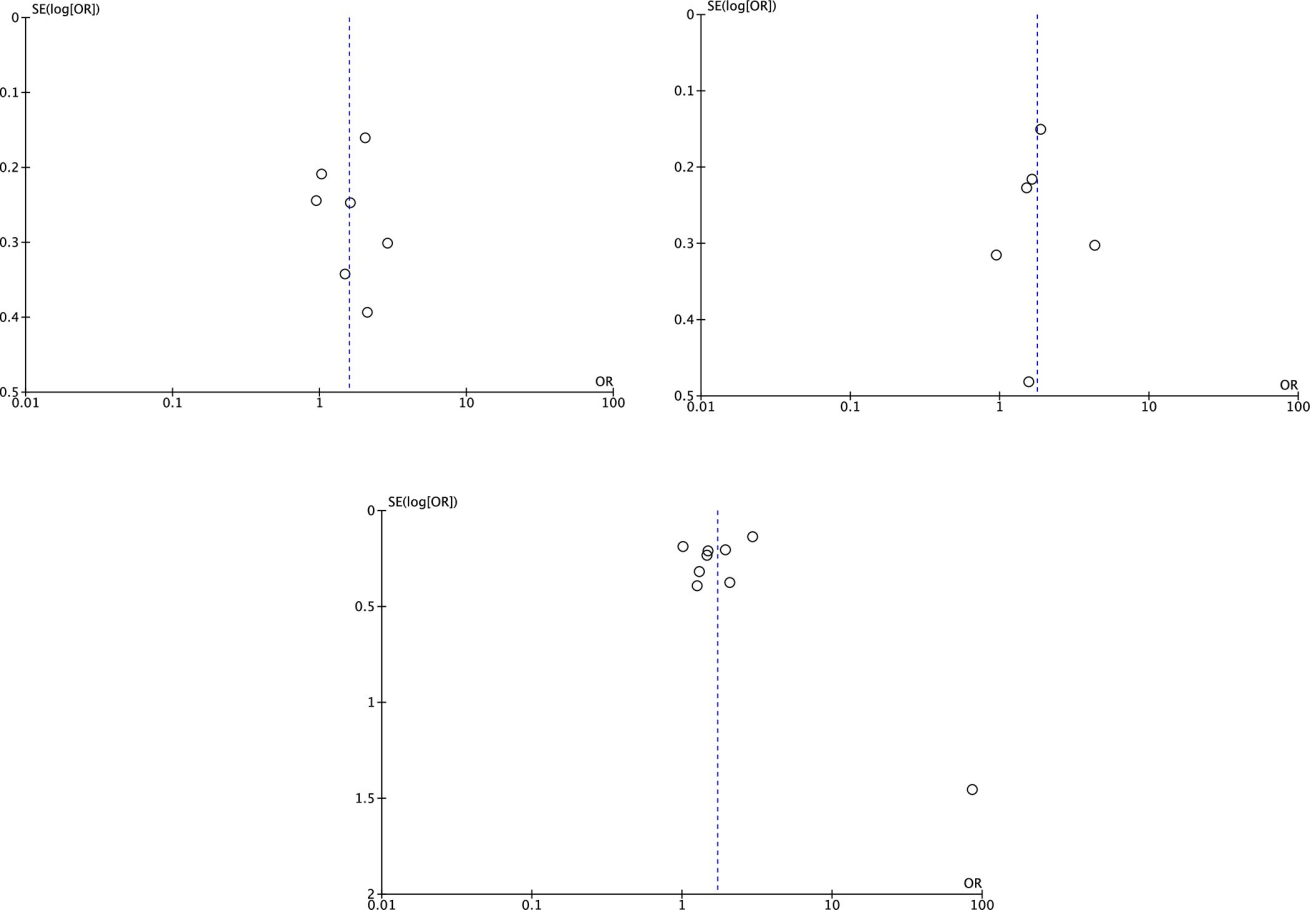
**Funnel plots of**. (a) knowledge of cancer screening, (b) Intention to Complete Cancer Screening, (c) Completion of Cancer Screening. OR: Odds ratio, SE: standard error, log: logarithm.

For each main outcome of interest, respective funnel plots were generated for evaluation of publication bias. The distribution of data points provided limited evidence for small study publication bias (Fig 10A–10C).

## Discussion

This study examined the effects of interventions on cancer screening among Chinese Americans. Outcomes investigated in this study included participants’ knowledge of cancer screening, intentions to complete cancer screening, and completions of cancer screening. Results showed that the interventions on cancer screening have a positive effect on all outcomes, including a 1.58, 1.78, 1.72 effect on knowledge of cancer screening, intentions to complete cancer screening, and completions of cancer screening, respectively, compared to the control group. In addition, subgroup analysis suggested individual-based, physician-led, and face-to-face interventions might be a good way to increase cancer screening among Chinese Americans, with the OR ranging from 1.82 to 2.83. To date, to our best knowledge, this is the first study examined the effects of evidence-based interventions on cancer screening among Chinese Americans. Findings from this study could potentially be used for developing sensitive intervention programs to increase cancer screening rates among Chinese Americans.

Utilizing appropriate intervention methods to expand Chinese Americans’ knowledge on cancer screening is effective in increasing cancer screening rates among this population. Previous studies have showed that lacking knowledge toward cancer screening is a barrier for Chinese Americans to obtaining the tests [46–48]. Chinese Americans’ lack of knowledge about the screening tests, availability of facilities that perform the tests as well as the extent of cost coverage act as barriers to screening. High-risk Chinese Americans’ lack of knowledge about cancer screening might be caused by the language barrier. Health care providers can help them by providing information about cancer screening and expanded insurance coverage. In addition, educational materials written in Chinese that provide resources on cancer screening to improve their knowledge would be necessary in improving their screening rates.

Furthermore, interventions which are both linguistically and culturally adapted to Chinese Americans seem effective in increasing cancer screening rates among this population. Seminars or counseling conducted in Mandarin or Cantonese, information materials written in fifth grade reading level Chinese [30], and visual media featuring Chinese cultural characteristics could help with the screening.

In addition, findings from the meta-analyses showed a physician-led, individual-based, direct in-person face-to-face client-focused intervention with multiple components could be the optimal way to enhance cancer screening uptake among Chinese Americans. Compared to the community worker or educator-led, group-based, clinician-focused, indirect remote or self-learning interventions with a single component, the aforementioned-methods have multiple strengths which could bring benefits to high-risk Chinese Americans. First, individual-based interventions could be more personally targeted. Sensitive and individually targeted information could be provided and discussed to overcome the language barrier which may exist in the group-based interventions. Second, compared to community workers or educators, physicians are widely trusted among Chinese Americans [49]. The level of trust-in-physicians among U.S. Chinese older adults was 42.0 out of 55 [49] on the Trust in Physician Scale [50]. By building a close rapport between physicians and high-risk Chinese Americans, interventions on cancer screening could be more effective. A physician-led intervention which aims to increase both the physicians’ and their patients’ knowledge levels of cancer screening could be a cost-effective way to increase cancer screening rates. Through the interventions on physicians, a larger portion of high-risk patients could be identified and reached. Patients who are eligible for cancer screenings could also be further recommended to receive screening by their physicians. Third, a direct in-person face-to-face intervention could provide opportunities for high-risk Chinese Americans to interact with the interveners. Any questions raised from the intervention could be answered immediately. Also, a direct in-person face-to-face intervention could help to facilitate relationships between interveners and high-risk Chinese American participants, which is beneficial to build rapport and further increase the uptake rate of cancer screening. Fourth, single component interventions are often insufficient to lead to sustainable change. On the contrary, multiple-component interventions not only affect the desired outcomes but also multiple associated outcomes [51]. It is necessary to have multiple components to address multi-level influences simultaneously, since multiple strategies are generally more effective than a single strategy for increasing cancer screening [52]. Lastly, compared to the clinician-focused intervention, the client-focused intervention was found to be more effective in increasing the completion rates of cancer screening among Chinese Americans. Multiple client-focused interventions, such as client reminders, one-on-one education, and group education should be implemented to help high-risk Chinese Americans to raise their awareness about screening cancers, increase their knowledge level about screening cancers, and overcome the barriers to screening cancers. However, strategies need to be efficient in developing multi-component interventions since such types of intervention could be labor- and cost-intensive.

Lastly, a trend of the intervention methods was noticed shifting from the video [32, 41, 42] or phone-based or -assisted [36, 40] interventions to in-person face to face educational seminars [33–35, 38, 44] from 2011 to 2018. Possible reasons for the trend may be related to the difficulties in assessing the effects of video or phone-based/assisted interventions, since participants’ utilization levels of the materials in the interventions are hard to evaluate; also, given that in person face to face interventions are more effective and easier to conduct than video or phone-based interventions, further technology development would be essential to utilize video or phone-based/assisted interventions.

## Limitations

This study has some limitations. First, as in all systematic reviews and meta-analyses, publication and other reporting biases may have affected our findings. Second, we found substantial heterogeneity among study effects, which diminishes the precision of our estimates for intervention effect sizes. We suspect this heterogeneity was due to the varied intervention methods, but I^2^ was only partially reduced by adjusting this factor. However, given the intervention categories in which all point estimates and virtually almost all limits of 95% CIs included clinically important, we are confident about the interventions’ benefit. Third, studies included in this meta-analysis were either good or fair quality studies; none of them were high quality studies given none of them met the three blinding criteria in the scale (blinding of the participants/therapists/assessors). As they were intervention studies which aimed to increase participants’ knowledge levels, participant and therapist blinding was not feasible, but blinding of the assessors was feasible given the design of the studies. Thus, in terms of the purpose of the studies, the insufficient strength of evidence reported in this review should not be interpreted as evidence that the interventions are not effective but, rather, as encouragement for additional research before effectiveness can be established.

## Conclusions

This systematic review and meta-analysis showed a statistically significant increase on participants’ knowledge of cancer screening, intentions to complete cancer screening, and completions of cancer screening with the implementation of cancer screening interventions. An individual-based, physician-led, and direct in-person face-to-face intervention method was suggested to be utilized in the cancer screening interventions. Future research programs and clinical practice which aim to increase the uptake rates of cancer screening among high-risk Chinese Americans should utilize language sensitive and individually targeted materials to increase this population’s knowledge levels of cancer screening; provide guidelines and aid service for physicians to initiate discussions around cancer screening; and offer in-person face-to-face opportunities for high-risk Chinese Americans to share their thoughts toward cancer screening, thus increasing this population’s intention to complete cancer screening and eventually increase the screening completion rate among this population. In addition, investigation about the trend of cancer screening uptake rates among the general Chinese American population is also necessary, which could help researchers and health care providers to better understand the status of cancer screening uptake among Chinese Americans.

## Supporting information

S1 Checklist PRISMA 2020 checklist. (DOCX)
